# Hypertrophic scar regression is linked to the occurrence of endothelial dysfunction

**DOI:** 10.1371/journal.pone.0176681

**Published:** 2017-05-04

**Authors:** Xi-Qiao Wang, Fei Song, Ying-Kai Liu

**Affiliations:** Burn Centre, Ruijin Hospital, Jiaotong University Medical School, Shanghai, P.R. China; IDI, Istituto Dermopatico dell'Immacolata, ITALY

## Abstract

Most microvessels have been shown to become stenosed or completely occluded during hypertrophic scar progression. Here, we examined the morphology of capillary endothelial cells (ECs) and fibroblasts using immunofluorescence staining for CD31 and alpha-smooth muscle actin (α-SMA) and electron microscopy. In addition, ECs and fibroblasts were isolated from scar tissues, and the levels of transforming growth factor beta 1 (TGF-β1), platelet-derived growth factor (PDGF), endothelin 1 (ET-1), vascular endothelial growth factor (VEGF) and basic fibroblast growth factor (bFGF) were assayed using ELISAs. Furthermore, we assessed cell viability, total collagen production, and cell apoptosis in hypertrophic scar-derived fibroblasts cultured with EC-conditioned medium. Then, anti-TGF-β1, anti-PDGF, anti-ET-1, anti-VEGF, and anti-bFGF neutralising antibodies were individually added to the EC medium to identify which growth factor plays a more important role in inhibiting fibroblasts biology. Our results showed microvessel lumen occlusion and EC atrophy during scar development, particularly in regressive scars (RSs). Additionally, EC growth factor secretion decreased and reached the lowest levels in RSs. Furthermore, based on the culture results, RS EC medium inhibited fibroblast viability and collagen production and induced apoptosis. Moreover, TGF-β1, PDGF, and bFGF played more important roles in these processes than VEGF and ET-1. The endothelial dysfunction occurring in hypertrophic scars contributes to fibroblast inhibition and scar regression, and reduced TGF-β1, PDGF, and bFGF levels play key roles during this process.

## Introduction

Hypertrophic scars caused by a burn, trauma, or surgery are accompanied by erythema, elevation, itching, and pain. After a certain period of hyperplasia, ranging from months to years, the scars cease to grow and spontaneously regress without treatment. Thus, some new factors, potentially scar formation inhibitors, may be secreted during this process. Activated fibroblasts and excess collagen production have been shown to play key roles in hypertrophic scar formation [1], whereas apoptosis within the scar tissue contributes to scar regression [2]. However, the underlying factors triggering fibroblast apoptosis and scar regression are unknown.

Fibroblast biology is generally regulated by the surrounding tissue microenvironment [3], and capillaries play a key role in establishing this environment. In the skin, capillaries are composed of a monolayer of endothelial cells (ECs), i.e., the endothelium, which harbours many channels that allow the passage of water, solutes, and macromolecules. Therefore, the endothelium is viewed as a modulatory interface between the microvessel lumen and neighbouring cells [4].

In addition, the endothelium is regarded as a massive endocrine organ that releases many types of growth factors, such as vascular endothelial growth factor (VEGF), platelet-derived growth factor (PDGF), transforming growth factor beta 1 (TGF-β1), and endothelin 1 (ET-1), thereby establishing the microenvironment [5–7]. Endothelium-derived growth factors are critical for regulating the development and maintenance of many organs, including the liver, pancreas, and nervous system [8–10]. Strong evidence supports a direct correlation between ECs and neurons in the brain [11]. Indeed, ECs not only exert a protective effect on neurons [12] but also initiate repair processes following injury and support neurite outgrowth by secreting shared growth factors [13, 14]. Therefore, vascular ECs play important roles in regulating cell biology and tissue homeostasis.

In humans, fibroblasts and vascular ECs, two major cell types within hypertrophic scars, communicate via nutrient transport and signal transmission. Based on the results of our previous study, microvessel occlusion and functional EC regression occur prior to scar regression [7]. However, the roles of this process in inducing EC dysfunction and subsequent scar regression are understudied. Therefore, our study aimed to understand EC behaviour during microvessel occlusion to determine whether ECs inhibit fibroblasts and regulate scar regression. Because developing hypertrophic scars usually undergo both hyperplastic and regressive phases, we classified scars as proliferative, regressive, or mature to investigate the dynamic morphological and biological changes in ECs and fibroblasts during scar development. Then, proliferative scar (PS)-derived fibroblasts were cultured with conditioned medium obtained from ECs from various scars to examine the effect of factors secreted by scar-derived ECs on fibroblast viability, total collagen production, and apoptosis.

## Materials and methods

### Human hypertrophic scar and normal skin (NS) sample collection

Hypertrophic scars were classified as proliferative, regressive, or mature based on their clinical appearance and wound healing duration [15]. PSs were characterised by deep redness, increased thickness, pruritus, and pain after 3 to 6 months of wound healing. Regressive scars (RSs) were defined characterised by a reduced thickness, pruritus, and pain after approximately 2 years of healing. Mature scars (MSs) were defined as flattened tissue with a near-NS appearance at or after 4 years of healing.

This study was approved by the local ethics committee of the Shanghai Jiaotong University Medical School and performed at the Burn Centre of Ruijin Hospital. We enrolled adults (age 18–50 years, average 34.3±5.2 years) who were admitted to our institution with hypertrophic scars (≥3 months) from January 2007 to January 2009; patients with keloids were excluded. Informed consent was obtained from 24 patients who agreed to participate in this study, and we certified that the use of all samples conformed to the ethical guidelines of the Declaration of Helsinki. Each group comprised 8 patients, from whom control samples were harvested from the NS adjacent to the scar margins.

### Assessment of morphological changes in scar tissue microvessels using immunofluorescence staining for CD31 and α-smooth muscle actin (α-SMA)

For immunofluorescence staining, sections of each sample were fixed with 4% paraformaldehyde for 30 min and then permeabilised with 0.2% Triton X-100 (T8787; Sigma-Aldrich, St. Louis, MO, USA) in phosphate-buffered saline (PBS) for 10 min. The sections were incubated with primary antibodies overnight at 4°C and then with secondary antibodies for 2 h at room temperature. The following primary antibodies were used: mouse anti-human CD31 (1:200, Cell Signaling Technology, Danvers, MA, USA) and rabbit anti-human α-SMA (1:200, Abcam, Cambridge, MA, USA). The following secondary antibodies were used: Alexa Fluor 488-conjugated donkey anti-mouse (1:200) and Alexa Fluor 594-conjugated donkey anti-rabbit (1:200) (both from Life Technologies, Shanghai, China). The nuclei were stained with 4’,6-diamidino-2-phenylindole (DAPI, H-1200; Vector Laboratories, Burlingame, CA, USA). Images of the immunofluorescence staining were captured using a confocal laser scanning microscope (Carl Zeiss, Oberkochen, Germany) using a 40x objective; six randomly selected optical fields were imaged from each sample, and a total of 48 fields were investigated (8 samples in each group). The green staining represents ECs and the red staining represents smooth muscle cells. The experiment was repeated three times and positive staining was identified by two other researchers who were blinded to the groups. The α-SMA expression levels were obtained by calculating the square of the percentage of positive cells using a Zeiss KS400 image analysis system (Carl Zeiss, Oberkochen, Germany). In addition, the open area of the microvessel lumen were also calculated.

### Assessment of microstructural changes in scar tissue ECs and fibroblasts using electron microscopy

Electron microscopy was employed as previously described to observe changes in the EC and fibroblast microstructures [7]. First, tissue samples were fixed with 2.5% glutaraldehyde followed by 1% OsO_4_. Then, the samples underwent serial dehydration, soaking, embedding in epoxy resin, and sectioning into ultra-thin 60-nm sections. The sections were stained with a solution of uranyl acetate and lead citrate, and then a transmission electron microscope (HITACH 500, Hitachi, Ltd., Tokyo, Japan) was used at a voltage of 75 kV to observe the microstructural changes in ECs and fibroblasts within the scar tissues. Two microvessels were captured in one section and a total of 16 microvessels from each group were investigated. In addition, 2 fibroblasts were from each section and 16 cells from each group were analysed. In addition, the size of both cell types were measured using HITACHI image processing software and then divided by the magnification to determine the practical cell size.

### Isolation of ECs and fibroblasts from hypertrophic scar and NS samples

Cells were isolated using a recently developed protocol from our laboratory [16]. Briefly, after removing the epidermis, the scar tissue or NS samples were minced into 1-mm^3^ pieces and sequentially digested with 0.5% type I collagenase and 0.2% trypsin solutions for 6 h and 2 h, respectively. Then, the digestion was stopped by adding 10% foetal calf serum (FCS) to neutralise the solution, and the samples were filtered through a 70-μm nylon mesh. The homogenates were centrifuged and resuspended in EC growth medium (Cascade Biologics, Portland, Oregon, USA). The cell-containing solutions were then seeded into flasks and cultured to confluence, after which the ECs were purified using CD105 magnetic bead columns (Miltenyi Biotec, Bergisch Gladbach, Germany). The CD105-positive cells were cultured in M131 medium supplemented with 10% FCS, whereas the CD105-negative cells were cultured in Dulbecco’s Modified Eagle’s Medium (DMEM) supplemented with 10% FCS. The cell types were characterised as ECs and fibroblasts, respectively. Subsequently, both cell types were cultured for 12 h. Then, the media was replaced with corresponding serum-free medium and the cells were cultured for an additional 6 h the conditioned media were collected for the ELISA assays and culture experiments.

### Quantification of TGF-β1, VEGF, PDGF, bFGF, and ET-1 production in ECs and fibroblasts using ELISAs

The conditioned media were collected from the cultures of both cell types isolated from different scar and NS samples and subjected to ELISAs according to the manufacturer’s instructions. Briefly, the different culture media were assayed in 96-well microplates, and the optical density (OD) values were measured at a wavelength of 405 nm using a ThermoMax microplate reader (Molecular Devices, Menlo Park, CA, USA). Serial dilutions (0–2,500 pg/ml) of recombinant human TGF-β1, VEGF, PDGF, bFGF, and ET-1 (Genzyme Corporation, Cambridge, MA, USA) were used to establish the respective standard curves. In addition, after collecting the conditioned media, the cells were detached from the plates and counted to quantify the amount of growth factors secreted per cell.

### Culture of proliferative fibroblasts in EC-conditioned medium

Fibroblasts isolated from PSs were cultured with the conditioned medium from ECs isolated from the different scar tissues described above for 48 h.

### Assessment of fibroblast viability following culture with EC-conditioned medium

Cell viability was quantified using an Alamar blue assay. Briefly, the cells in each well were washed twice with PBS and incubated with 150 μl of Alamar blue solution (1 μl of Alamar blue dye mixed with 99 μl of DMEM) per well at 37°C for 1 h. Then, 100 μl of the suspension were transferred into a 96-well plate to determine the fluorescence using a POLARstar OPTIMA microplate system (Optima, BMG Lab Technologies, Germany).

### Quantification of the number of apoptotic fibroblasts following culture with EC-conditioned medium using the TUNEL assay

Fibroblasts were seeded into 8-well chamber slides and cultured with EC-conditioned medium for 48 h before each chamber was assessed using the TUNEL assay. The cells were fixed with 4% paraformaldehyde, washed once with PBS, and then permeabilised with 0.2% Triton X-100/PBS. After further washes in PBS, the cells were incubated with 50 μl of the TUNEL reaction mixture for 60 min at 37°C. After incubation with the TUNEL reaction mixture, the slides were washed with PBS, mounted under glass coverslips using the SlowFade Gold anti-fade reagent (Invitrogen, Victoria, Australia) containing DAPI, and sealed with clear nail polish. A Nikon Eclipse TE2000-U fluorescence microscope was used to visualise and image the cells. The positive staining was assessed by two other researchers who were blinded to the groups. The number of apoptotic cells was calculated from an average of 6 optical fields per sample and expressed as a percentage of the total cells.

### Quantification of total collagen production by fibroblasts cultured in EC-conditioned medium

Total collagen levels were quantified using a Sirius red collagen assay. The cells were incubated with 0.1% Sirius red (Sigma-Aldrich, St. Louis, MO, USA) for 90 min, washed with water, and dried overnight. Then, 200 μl of 0.1 M NaOH were added to each well, and the plates were placed on a shaker for 1–2 min. Then, 50 μl of the supernatant from each well were transferred into a 96-well plate to measure fluorescence using a POLARstar OPTIMA microplate system. Images of the Sirius red-stained cells were acquired using digital photography (Nikon Coolpix 4500, Nikon, Japan).

### Identification of the EC-derived growth factors that affected fibroblast biology using antibody-mediated competition in fibroblasts cultured with normal EC-conditioned medium

Anti-TGF-β1, anti-PDGF, anti-ET-1, anti-VEGF, and anti-bFGF neutralising antibodies were used individually to block the effects of their corresponding growth factors in the EC-conditioned medium that had been added to the fibroblast cultures to determine which EC-derived growth factors affected fibroblast biology. The control group was supplemented with 0.9% NaCl. After 48 h of culture, cell viability, total collagen production, and apoptosis were determined.

### Statistical analyses

All data are presented as means ± standard deviation. Statistical significance was determined using a one-way analysis of variance followed by Student’s t-test. P values ≤0.05 were considered significant.

## Results

### Microvessels and fibroblasts exhibit dynamic changes in scar tissue

**Fig 1:**
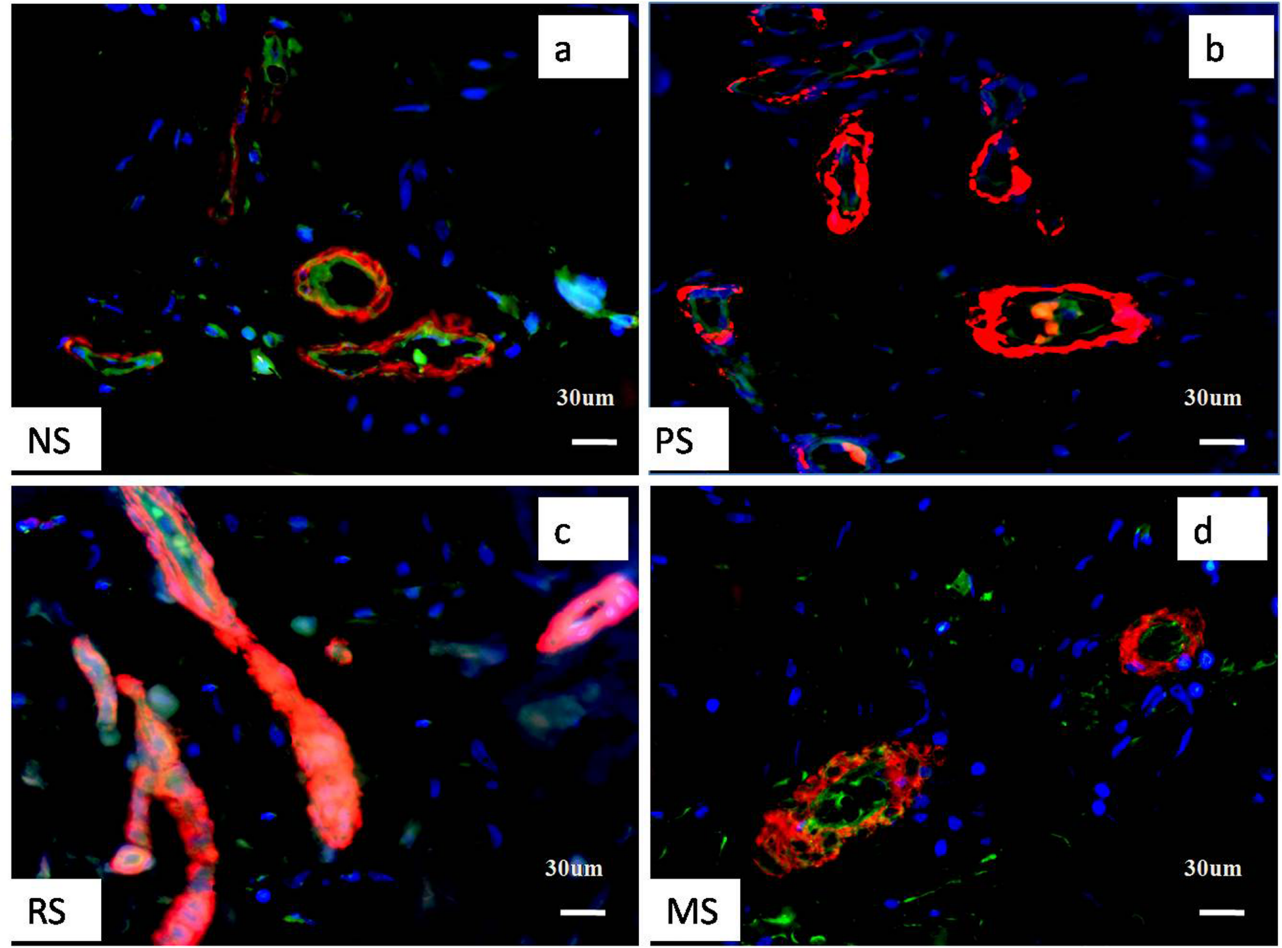
Pathological changes in hypertrophic scar tissue at different stages were shown by immunofluorescence staining for CD31 and α-SMA. Although few microvessels and fibroblasts were observed in NS, more were observed in PSs; however, most of the microvessels were partially or completely occluded in RSs, and the RSs exhibited fewer fibroblasts. In MSs, the numbers of microvessels and cells were comparable to those in NS. a, b, c, and d represent NS, PS, RS and MS samples, respectively. Scale bar: 30 μm. The green staining represents ECs, the red staining represents smooth muscle cells, and the blue staining represents fibroblasts.

CD31 and α-SMA were used to label ECs (green colour) and smooth muscle cells (or myofibroblasts) (red colour), respectively, to examine the relationship between microvessels and tissue fibroblasts. Immunofluorescence staining revealed that both proteins were expressed around microvessels, and most negative staining was observed in fibroblasts located outside the microvessel. A few microvessels were observed in NS tissue; however, a marked increase in the number of microvessels was observed in PSs, but little CD31 staining was observed, perhaps due to of the substantial increase in α-SMA expression. In RSs, most vessels were completely occluded, α-SMA was expressed at high levels, and the CD31 staining was almost obscured by the α-SMA staining (pink colour). Conversely, in MSs, the number of microvessels was reduced. The change in the number of vessels was fairly consistent with our previous study [17]. We calculated the percentage of α-SMA-expressing cells and observed a significant increase in PSs compared to the percentage in NS (28.9±3.3% versus 10.1±1. 8%, P<0.05), which was further elevated in RSs (42.7±6.2%, P<0.05). This value was reduced in MSs and comparable to that in NS (9.1±1.5%, P>0.05). However, the open area of microvessel lumen was markedly increased in PSs compared to that in NS (351.1±41. 8 μm^2^ versus 120.9±22.3 μm^2^, P<0.05), but substantially reduced in RSs (41±6.8 μm^2^, P<0.05. Fig 1A–1D), which is likely correlated with the compression of the increased number of smooth muscle cells.

In our previous study, we observed moderate hypoxia in PS, which likely promotes myofibroblast proliferation and microvessel formation, and severe hypoxia in RS [16], which perhaps further promotes myofibroblast proliferation but damages ECs.

### EC and fibroblast morphology exhibit dynamic changes in scar tissues

**Fig 2:**
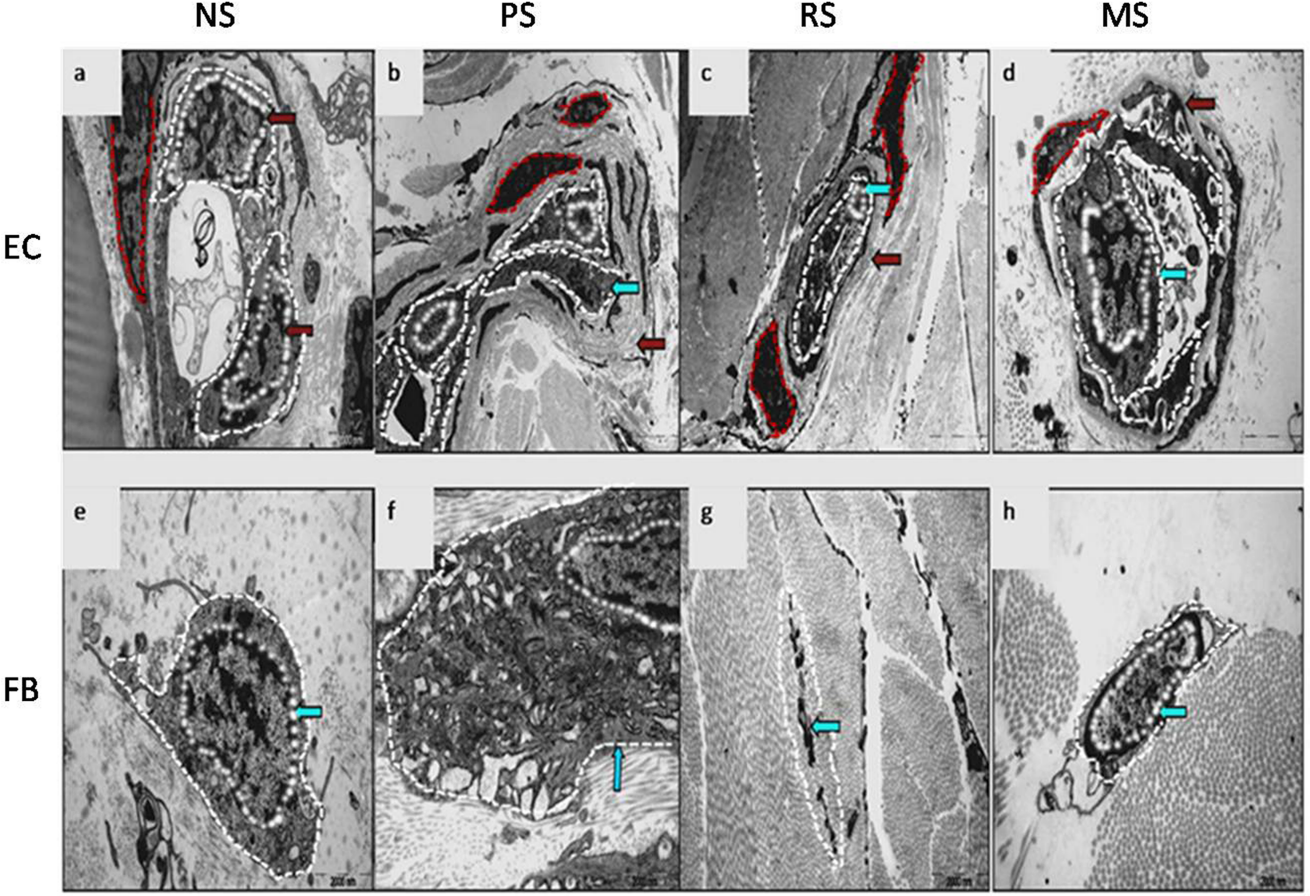
Electron microscopy examination of human hypertrophic scars at different stages. Morphological changes in the microvessels and ECs in NS (a, ×9,700), PS (b, ×4,200), RS (c, ×5,800) and MS samples (d, ×13,500) are shown. The blue arrow indicates ECs and the red arrow indicates the basement membrane. The white dotted line shows ECs, and the red dotted line shows smooth muscle cells. Morphological changes in the fibroblasts in NS (e, ×13,500), PS (f, ×17,500), RS (g, ×13,500) and MS samples (h, ×13,500) are shown. The blue arrow indicates fibroblasts.

According to the electron microscopy examination, the vascular lumen was open, with 1–2 ECs containing an abundant cytoplasm, and the cell size was approximately 0.45±0.09 μm^2^ in NS (cross section, Fig 2A). The NS basement membrane was not distinguished in our study, probably due to tissue-specific properties. In PSs, the microvascular lumen was partially occluded, with 3–4 ECs exhibiting an irregular morphology (Fig 2B), and the cells showed a marked reduction in size (0.33±0.07 μm^2^, P<0.05). In these samples, the basement membrane was replaced with thick collagen fibres. In RSs, the microvascular lumen was almost completely occluded, and the ECs were atrophied and surrounded by a very thick collagen layer (Fig 2C); in addition, the cell size was significantly reduced compared with that in the normal control (0.21±0.06 μm^2^, P<0.05). However, in MSs, the microvascular lumen was reopened, the ECs displayed a relatively abundant cytoplasm, a clearly defined basement membrane structure was observed (Fig 2D), and the cell size was comparable to normal ECs (0.40±0.08 μm^2^).

The electron microscopy examination revealed that NS fibroblasts contained an abundant cytoplasm and were 1.3±0.2 μm^2^ in size on average (Fig 2E). In PSs, the number of cytoplasmic organelles was significantly increased, the endoplasmic reticulum was enlarged (Fig 2F), and the cell size was significantly increased (4.1±0.3 μm^2^, P<0.05). In RSs, some cells were unusually disintegrated, with an appearance similar to apoptotic bodies (Fig 2G). In MSs, the cell size and cytoplasmic content had generally returned to normal levels (Fig 2H).

### TGF-β1, VEGF, PDGF, ET-1, and bFGF secretion is reduced in ECs but increased in fibroblasts during scar progression

**Fig 3:**
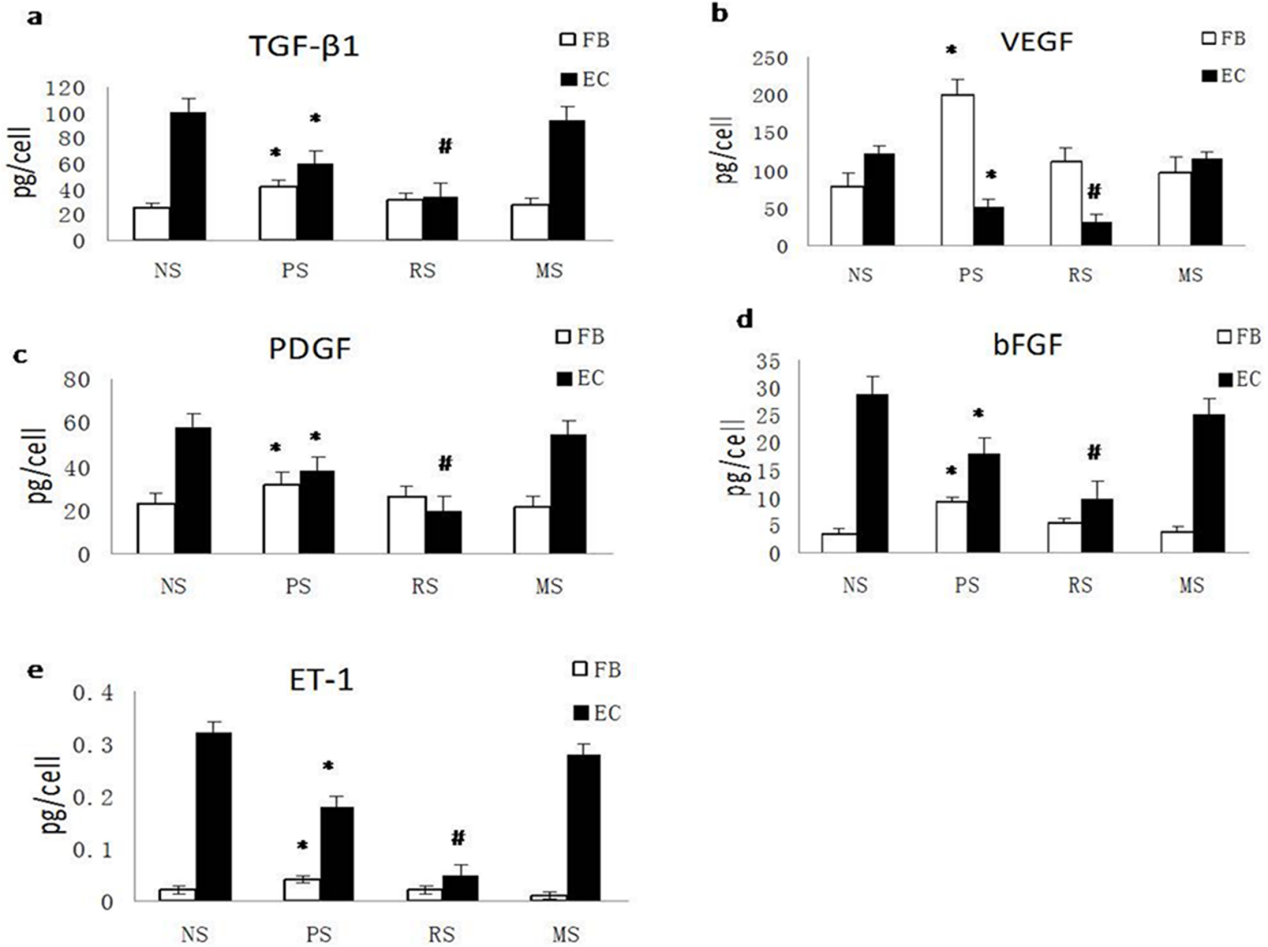
TGF-β1, VEGF, PDGF, ET-1, and bFGF secretion is suppressed in ECs but increased in fibroblasts during scar development. A. Quantification of the secretion of TGF-β1 (a), VEGF (b), PDGF (c), bFGF (d), and ET-1 (e) from fibroblasts and ECs from NS, PS, RS and MS. NS, PS, RS and MS represent normal skin, proliferative scar, regressive scar and mature scar, respectively. * P<0.05, # P<0.01, n = 8.

The cells isolated from scar and NS tissues were cultured and the conditioned cell culture media were harvested to quantify growth factor levels using ELISAs. High levels of the TGF-β1, VEGF, PDGF, ET-1 and bFGF proteins were produced by normal ECs; however, these levels were significantly decreased in PSs (P<0.05) and reached the lowest level in RSs (P<0.01). In MSs, growth factor levels were comparable to those in NS (P>0.05, Fig 3A–3E). However, in fibroblasts, the levels of these factors were significantly increased in PSs. Although the levels were sharply reduced in RSs, they remained higher than in NS controls, but the differences were not significant (P>0.05). In MSs, the levels of these factors almost returned to NS levels (Fig 3A–3E).

Based on these data, ECs produced much more TGF-β1, PDGF, ET-1, and bFGF, but not VEGF, than fibroblasts, which could provide a major source of growth factors for biological processes in fibroblasts. Conditions causing a reduction in the levels of these factors may exert a negative effect on fibroblast biology.

### Conditioned medium obtained from cultured ECs from hypertrophic scars inhibits fibroblast viability

**Fig 4:**
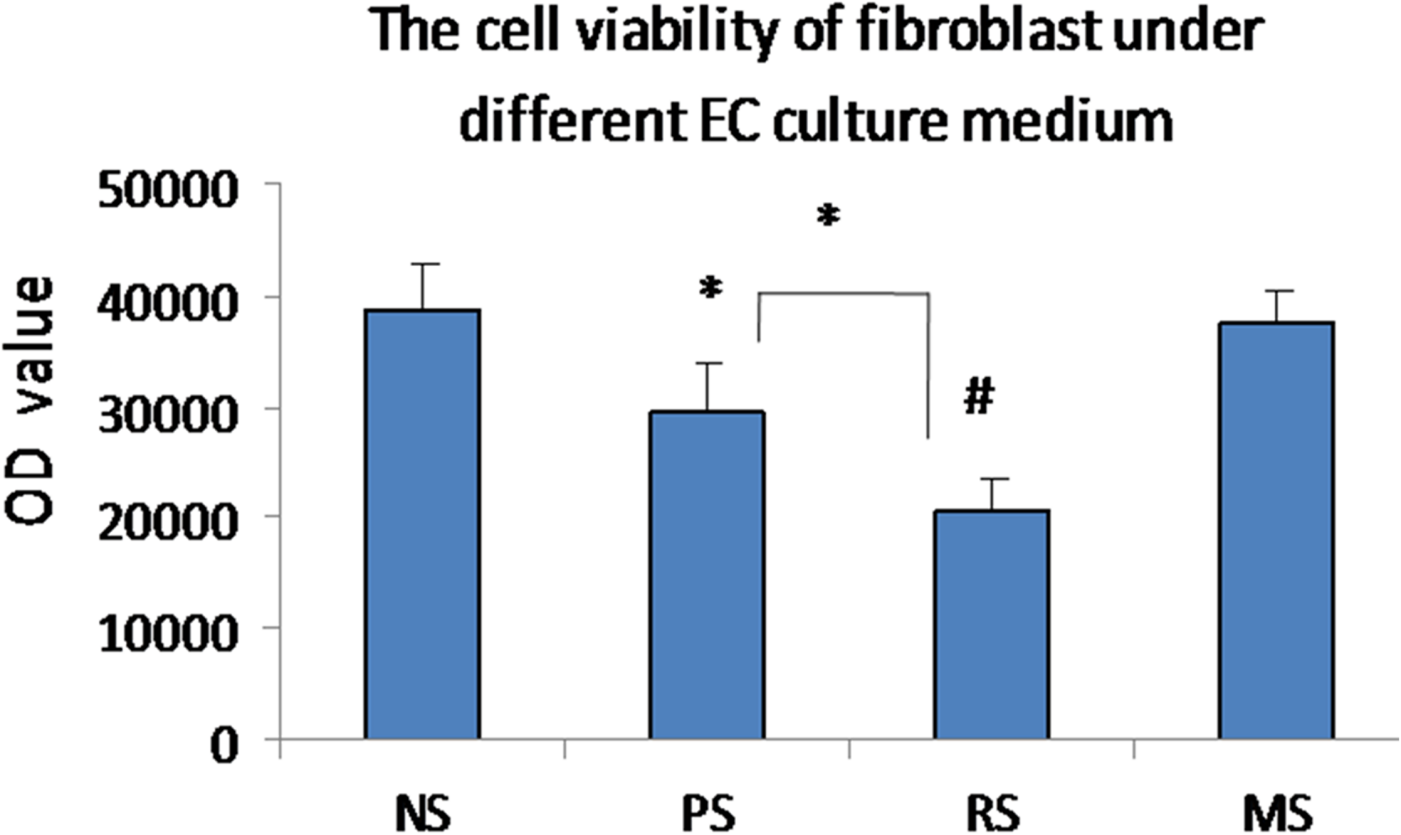
EC-conditioned media inhibit cell viability. Quantification of fibroblast viability when cultured with NS, PS, RS and MS EC-conditioned media. NS, PS, RS and MS represent normal skin, proliferative scar, regressive scar and mature scar, respectively. * P<0.05, # P<0.01, n = 8.

Fibroblasts were cultured with the conditioned medium collected from cultured ECs from various scars to assess the effect of ECs on fibroblast viability. Fibroblast viability was maintained at a certain level when the cells were cultured with normal EC-conditioned medium (38,712±427) but decreased when fibroblasts were cultured with PS EC-conditioned medium (29,538±317, P<0.05) and reached the lowest level when the cells were cultured with RS EC-conditioned medium (20,385±247, P<0.01). A significant difference in fibroblast viability was observed between the PS and RS EC-conditioned media (P<0.05). When fibroblasts were grown in MS EC-conditioned medium, viability increased to levels comparable to the levels observed when the cells were grown in the control NS EC-conditioned medium (Fig 4). Based on these results, fibroblast viability gradually decreases during scar development, reaching its lowest level in RSs.

### Conditioned medium from cultured ECs from hypertrophic scars induces fibroblast apoptosis

**Fig 5:**
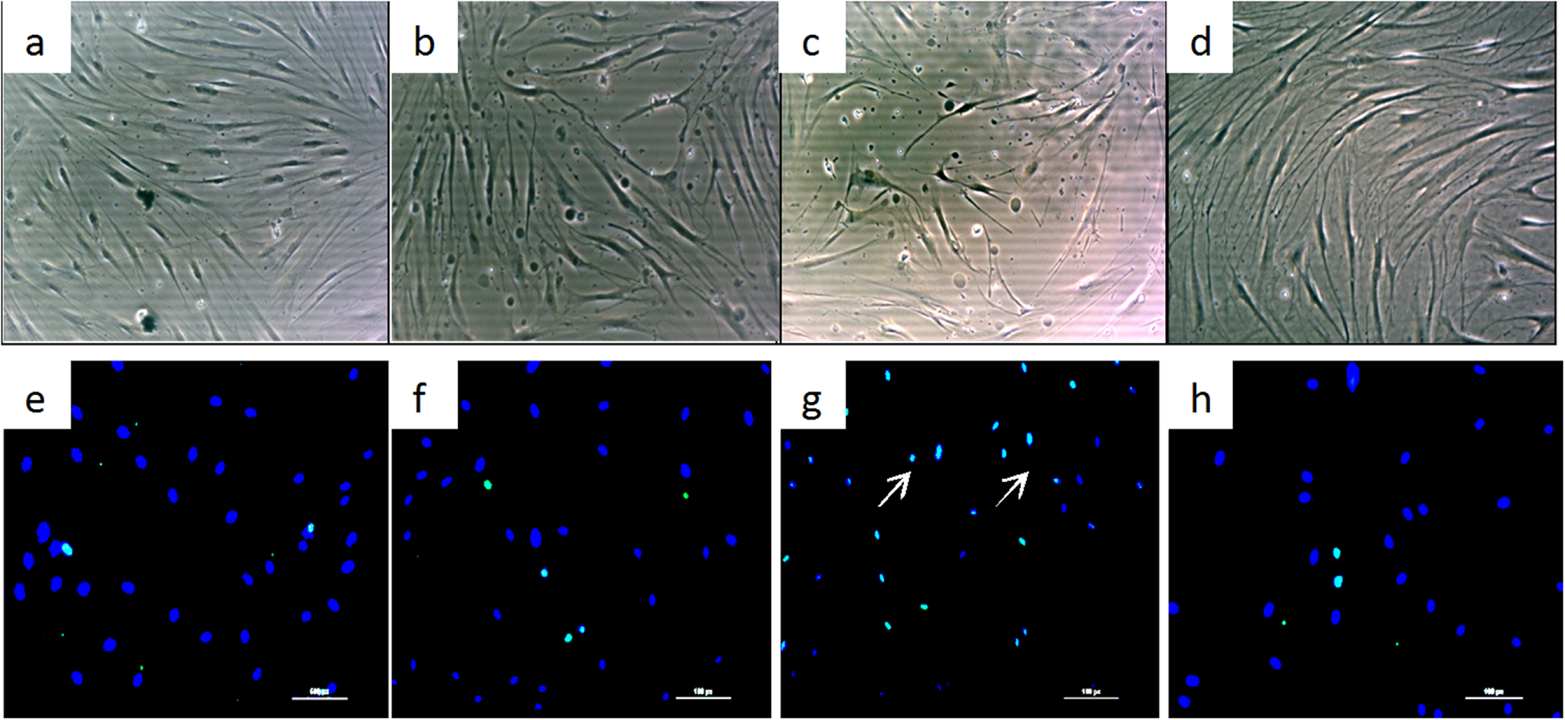
EC-conditioned media induce cell apoptosis. The morphology of fibroblasts was imaged under a light microscope after culture with NS (a), PS (b), RS (c) and MS EC-conditioned media (d). Fibroblast apoptosis was viewed using immunofluorescence staining after co-culture with NS (e), PS (f), RS (g) and MS EC-conditioned media (h). Apoptotic nuclei were stained green and are indicated by arrows, whereas negative nuclei were stained blue. NS, PS, RS and MS represent normal skin, proliferative scar, regressive scar and mature scar, respectively. The scale bar represents 50 μm, n = 5.

Cell morphology and apoptosis were assessed *in vitro* after culturing fibroblasts in conditioned media obtained from cultured ECs from various scars. Fibroblasts cultured in RS EC-conditioned medium exhibited unhealthy morphological features and fewer cells under the light microscope compared with cells cultured in NS EC-conditioned medium. More importantly, some dead cells were observed when RS EC-conditioned medium was used (Fig 5A–5D). Immunofluorescence staining showed minimal evidence of cell apoptosis in the normal control group (1.4±0.4%, Fig 5E), without any evident increase upon culture with PS EC-conditioned medium (2.2±0.8%, P>0.05, Fig 5F). However, fibroblast apoptosis increased significantly when the cells were cultured in RS EC-conditioned medium (13.2±4.1%, P<0.01, Fig 5G) and returned to approximately normal levels when cultured in MS EC-conditioned medium (P>0.05, Fig 5H)). Thus, the EC-conditioned medium from samples progressing along the scar development stages exerted an inhibitory effect on fibroblasts and even induced apoptosis when the conditioned medium was obtained from ECs reaching the scar regression stage.

### Conditioned medium from cultured ECs from hypertrophic scars inhibits total collagen production in fibroblasts

**Fig 6:**
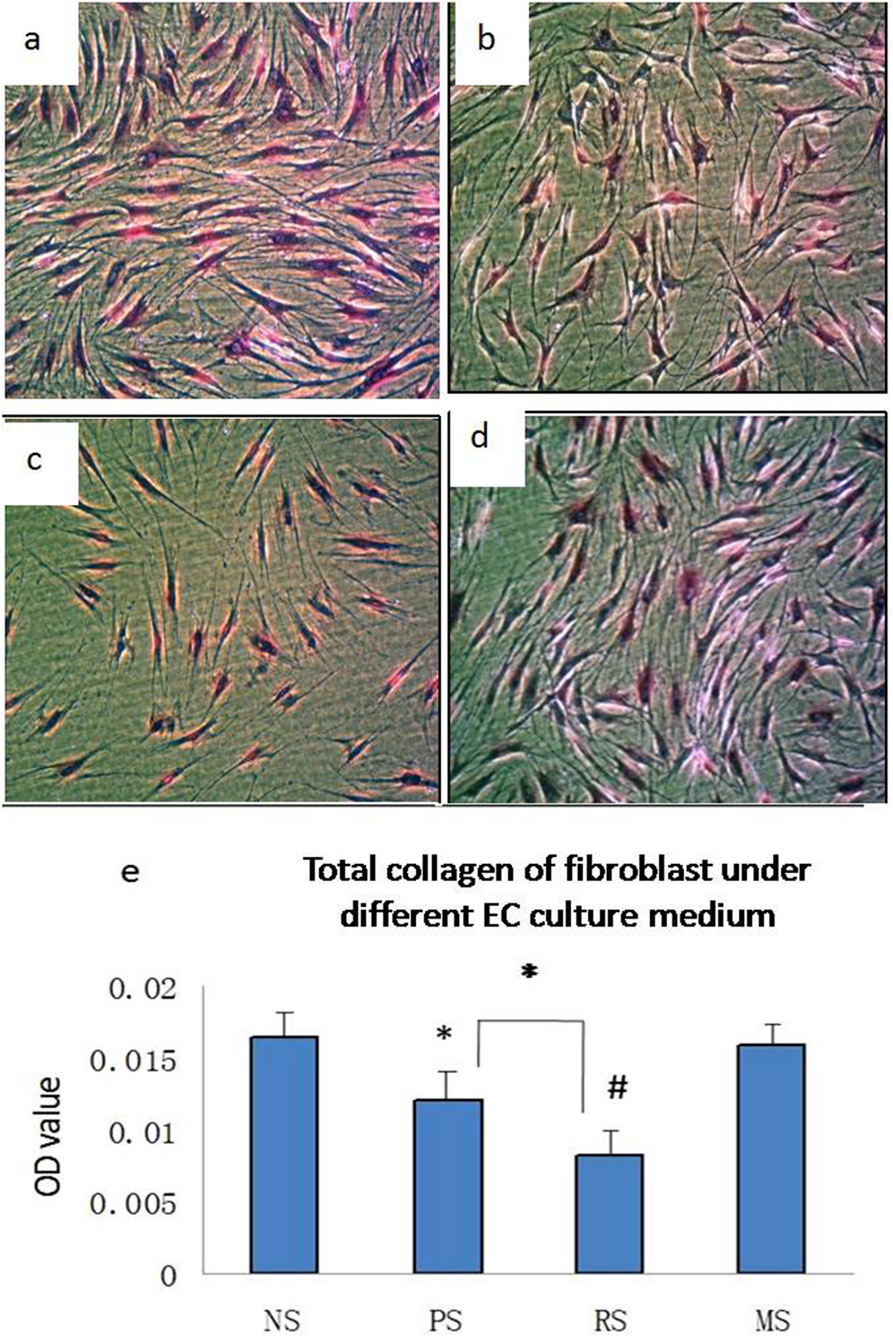
EC-conditioned media inhibit collagen production. Images of Sirius red-stained fibroblasts after culture with NS (a, 400x), PS (b, 400x), RS (c, 400x) and MS EC-conditioned media (d, 400x). The quantification of total collagen production by fibroblasts after culture with the various EC-conditioned media is shown (e). * P<0.05, # P<0.01, n = 8.

Sirius red assays were used to quantify total collagen production in fibroblasts cultured with conditioned medium obtained from cultured EC from various scars. Fibroblasts produced large amounts of total collagen when cultured with NS EC-conditioned medium (0.017±0.002, Fig 6A), and collagen levels tended to decrease gradually when cultured with either PS or RS EC-conditioned media, although both conditions yielded significantly different collagen levels than the levels observed under the control culture conditions (0.012±0.002, P<0.05; 0.008±0.001, P<0.01, Fig 6B and 6C). A significant difference in the collagen levels produced by fibroblasts cultured with PS and RS EC-conditioned media was observed (P<0.05). Total collagen production returned to normal levels when fibroblasts were cultured with MS EC-conditioned medium (P>0.05; Fig 6). Thus, total collagen production also gradually decreases during scar development, reaching its lowest level in RSs.

### Reduced levels of EC-derived TGF-β1, PDGF, and bFGF play more important roles than VEGF and bFGF in regulating fibroblast biology

**Fig 7:**
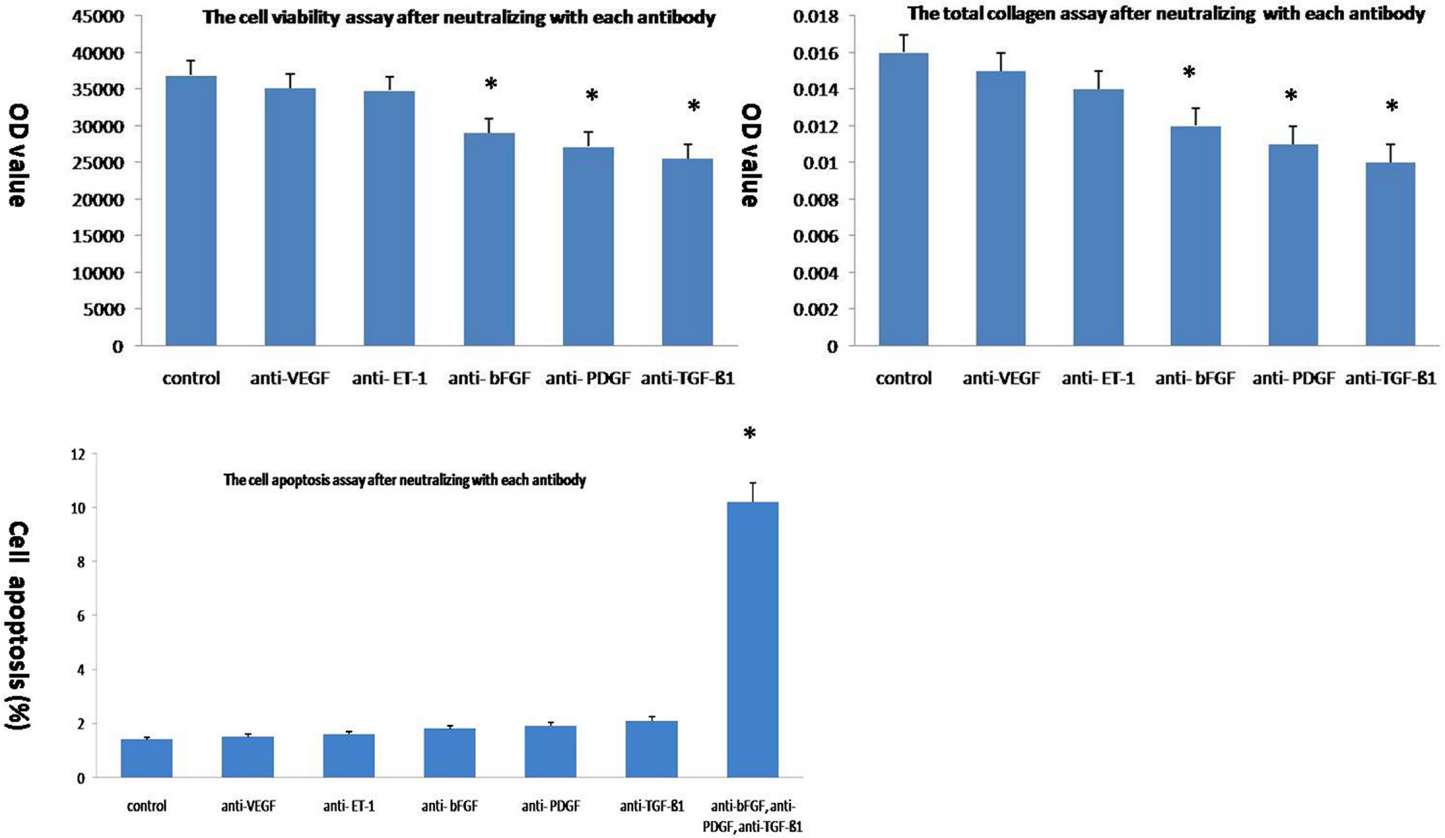
Reductions in the levels of EC-derived TGF-β1, PDGF, and ET-1 play more important roles than reductions in VEGF and bFGF levels in regulating fibroblast biology. Neutralising antibodies against TGF-β1, VEGF, PDGF, ET-1, and bFGF were individually added to EC-conditioned medium, and then the resulting cell viability (a), total collagen production (b), and numbers of apoptotic cells (c) were determined.

We individually added neutralising antibodies against each growth factor to normal EC-conditioned medium and assayed fibroblast viability and total collagen production after 48 h of culture to determine which of the secreted growth factors among TGF-β1, PDGF, ET-1, VEGF, and bFGF played more important roles in fibroblast biology. The viability of fibroblasts grown in the presence of anti-TGF-β1, anti-PDGF, or anti-bFGF antibodies was significantly reduced compared with that of cells grown in the presence of the isotype control (25,567±313, 27,165±366, 29,072±398, respectively, versus 36,892±434; P<0.05, Fig 7A). Conversely, the effects of the anti-VEGF and anti-ET-1 antibodies were not significant compared to the controls (35,089±440 and 34,796±411, respectively, versus 36,892±434 P>0.05). Similarly, total collagen production was also significantly reduced in the presence of anti-TGF-β1, anti-PDGF, and anti-bFGF neutralising antibodies (0.021±0.002, 0.019±0.002, and 0.018±0.002, respectively, versus 0.016±0.002, P<0.05, Fig 7B). Similar to their effects on cell viability, the anti-VEGF and anti-ET-1 antibodies did not produce a significant difference in collagen production. Although none of the individual blocking antibodies affected apoptosis, the combination of anti-TGF-β1, anti-PDGF, and anti-bFGF antibodies promoted apoptosis significantly (10.2±2.1 versus 1.4±0.4, P<0.05, Fig 7C).

### Discussion

The activation of keloid fibroblasts by neovascular ECs was shown to be the trigger for the initiation of fibrotic reactions [18]. However, the interaction between capillaries and fibroblasts during the development of hypertrophic scars has attracted less attention[S1 Fig].

During scar development, the microvessel lumen becomes occluded, ECs become atrophied and the basement membrane thickens. In addition, growth factor secretion by ECs decreases. Impairments in the ability of ECs to maintain vascular homeostasis is referred to as “endothelial dysfunction” [19, 20], which is associated with many diseases and comprises many aspects of endothelial physiology, including a reduction in cytokine secretion, a disruption in the balance between vessel constriction and dilation, and a change in vessel permeability [21, 22]. Therefore, in our study, the structural and biological changes observed in the endothelium indicated that endothelial dysfunction was present in human hypertrophic scars.

The positive effects of TGF-β1, PDGF, and ET-1 on fibroblast proliferation and collagen production are well known [23–28], whereas VEGF, bFGF and ET-1 are known for their pro-angiogenic properties [29,30]. Both of these roles contribute to the formation of hypertrophic scars in humans. However, during scar development, the levels of these growth factors were reduced in the cultured ECs obtained from the scars and reached their lowest expression levels in RSs, resulting in a significant inhibitory effect on fibroblast viability and total collagen production while triggering apoptosis in scars at this stage. During the regression process, the reduction in TGF-β1, PDGF, and bFGF levels plays a more important role and synergistically inhibits fibroblasts. Of course, other, perhaps less commonly known factors may be involved in this process and will be investigated in the future. Therefore, the reduction in growth factor secretion by ECs represents one aspect of endothelial dysfunction leading to scar regression.

However, the results observed using the PS EC-conditioned medium in the culture experiments, i.e., reduced viability and collagen production, were inconsistent with the results of a previous study in which fibroblasts in hypertrophic scars were hyperactive [15]. Oher factors, such as hypoxia, which stimulate scar formation [17] and promote fibroblast activation [31, 32], may counteract the effects of endothelial dysfunction. Therefore, slight endothelial dysfunction may not completely inhibit fibroblast activation alone, and a certain dysfunction threshold is required to decrease cell viability and trigger apoptosis.

In addition, during scar development, the basement membrane was replaced by a very thick collagen layer. Based on the rounded shape of the collagen layer, we inferred that this structure was likely produced by smooth muscle cells [33], which increased in number during scar formation. As the basement membrane thickens, it blocks EC-derived growth factors from passing through the membrane, further reducing cell nutrition, inhibiting fibroblast activity and contributing to scar regression.

Why does endothelial dysfunction occur in scar tissue? During scar development, the large collagen deposits around the microvasculature and the strong smooth muscle cells around the ECs are postulated to mechanically constrict the microvessels, leading to both their occlusion and cellular damage. Therefore, smooth muscle cells likely play important roles during this process.

Limitations of this study include the fact that patient enrolment was constrained by our burn centre, and thus a limited number of patients was available for investigation. In addition, the collagen production assay could be improved by counting the number of cells and quantifying the amount of collagen in each cell.

In conclusion, endothelial dysfunction occurring during the scar development process decreased the viability and collagen production capacity of fibroblasts, which are associated with scar regression. Our results not only provide a deeper understanding of scar regression but also provide insights into a novel strategy for the treatment of hypertrophic scars. Our results are also consistent with the results reported by Zhang et al., who achieved reduced scar formation after healing by pre-treating wounds with the angiogenesis inhibitor angiostatin [34]. Similarly, our team observed scar regression [35] after treating hypertrophic scars on rabbit ears with Endostar, a modified recombinant human endostatin that inhibits EC function. Together with our results, these successful treatments also suggest that endothelial dysfunction occurs in scar tissue.

## Supporting information

S1 Fig The proposed model of the tissue microenviroment with EC and fibroblast. (TIF)
