# Supplementary figures and images for: Hypertrophic scar regression is linked to the occurrence of endothelial dysfunction
Source: PLoS One. 2017 May 4;12(5):e0176681. doi: 10.1371/journal.pone.0176681 (PMC5417599; doi:10.1371/journal.pone.0176681)

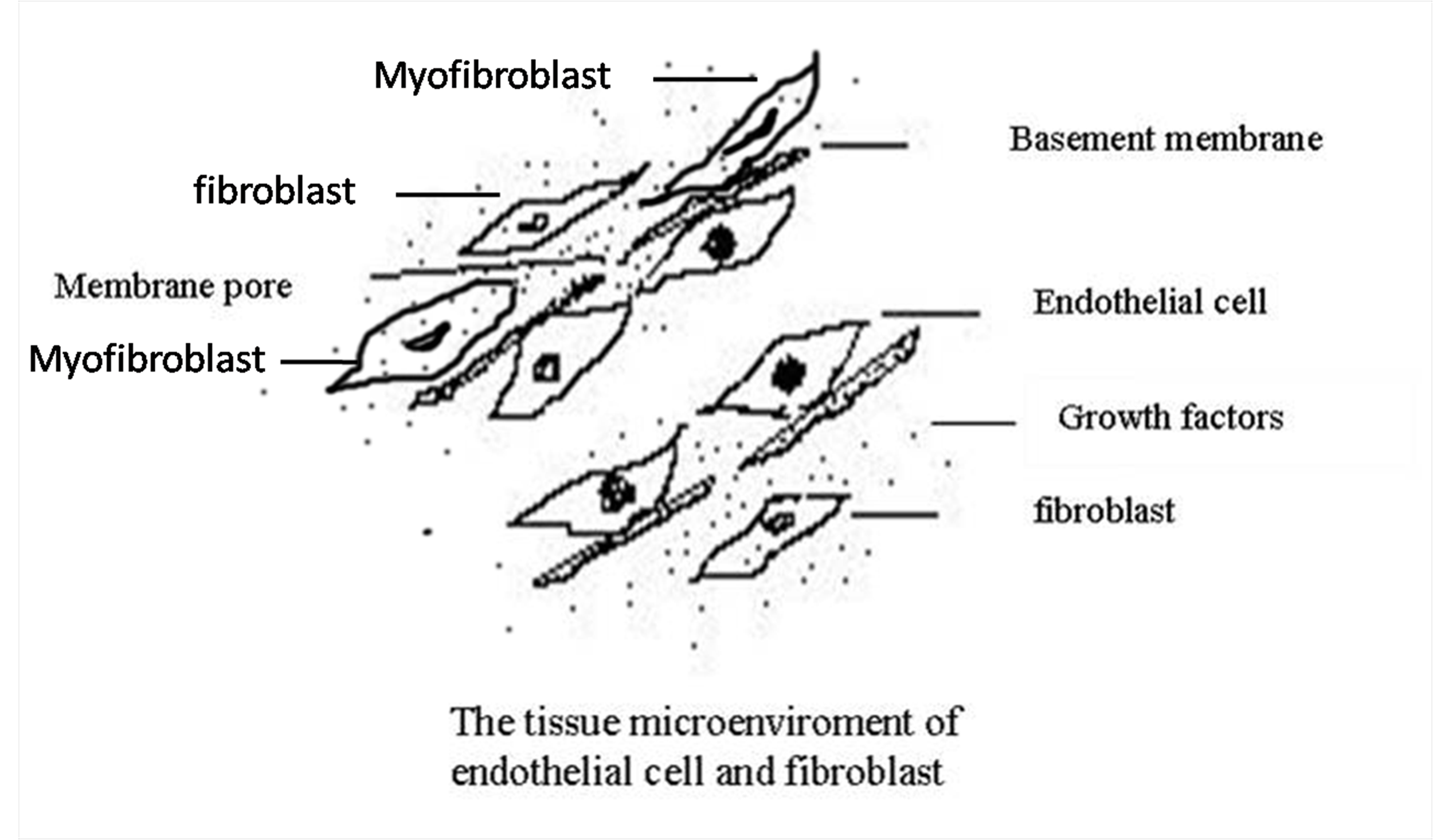

Supplement: S1 Fig — (TIF) [file pone.0176681.s001.tif]
